# Homogenization Reveals Large-Scale Dynamics in the Spread of Chronic Wasting Disease

**DOI:** 10.1007/s11538-025-01456-8

**Published:** 2025-05-20

**Authors:** Jen McClure, James Powell

**Affiliations:** Department of Mathematics and Statistics, Utah State University, Logan, UT 84341 USA

## Abstract

Thresholds in environmental transmission can significantly alter the dynamics of disease spread in wildlife. However, the impact of thresholds in landscapes with high spatial variability is not well understood. We investigate this phenomenon in chronic wasting disease (CWD), a degenerative cervid illness exhibiting direct transmission between individuals and indirect transmission through environmental hazard. The indirect pathway exhibits threshold behavior analogous to a strong Allee effect. We derive a partial differential equation (PDE) model for CWD on the scale of hours and tens of meters. Leveraging highly variable landscape structure, we homogenize this model to yield an asymptotically accurate approximal model on the scale of years and kilometers. Our homogenized model describes the aggregate effect of thresholded transmission on large scales – to our knowledge, the first time such a description has been identified. The model predicts that direct transmission in CWD will lead to pulled fronts, whereas indirect transmission generates pushed fronts. Pushed fronts allow CWD to spread even when infectives infect less than one susceptible on average. We use a hypothetical binary distribution of habitat types to showcase the homogenized model’s ability to predict how distribution of cover in a landscape can influence CWD spread and potential mitigation efforts.

## Introduction

A broad class of wildlife diseases spread between hosts via multiple transmission pathways (Roberts et al. 2021; Loh et al. 2015; Lange et al. 2016). Perhaps the most basic categorization of pathways is to separate direct transmission between hosts and indirect transmission from environmental sources such as surfaces, water, or aerosols. Although some diseases are suitably modeled using just a direct transmission pathway (Breban 2013), indirect transmission must be explicitly considered in cases when the pathogen persists for a long time in the environment or when infection requires exposure to a threshold dose of pathogen (Joh et al. 2009).

Chronic Wasting Disease (CWD) presents a salient case study for the relative importance of direct and indirect transmission. CWD is a transmissible spongiform encephelopathy affecting several cervid species including deer (genus *Odocoileus*), elk (*Cervus canadensis*), moose (*Alces alces*), and caribou (*Rangifer tarandus*). Since the disease was identified in 1967 its distribution has rapidly expanded to hundreds of counties in the United States and Canada, as well as South Korea and the Scandinavian peninsula (Haley and Hoover 2015). Increased prevalence has stoked mounting public concern over CWD’s impact on ecosystems (Escobar et al. 2020) and the hunting industry (Erickson et al. 2019; Schroeder et al. 2022), as well as its possible transmission to other species, including humans (Belay et al. 2004). As CWD continues to spread, it is crucial to understand the relative importance of direct and indirect transmission. Modeling efforts can help identify the factors most relevant to management efforts and likely ecological impacts.

The infectious agent of CWD is a prion, a misfolded version of a naturally-occurring protein in the host’s central nervous system. The folding of prions is energetically favorable, and thereby capable of converting healthy proteins into more prions on contact (Haley and Hoover 2015). CWD can be spread through direct or indirect transmission pathways – the latter of which have gained considerable attention (Miller et al. 2004; Almberg et al. 2011; Cortez and Weitz 2013; Vasilyeva et al. 2015; Thompson et al. 2024). CWD-infected deer shed prions in their blood, saliva, urine, and feces. Many of these bodily fluids contains infectious levels of prion load (Mathiason et al. 2006; Haley et al. 2009; Bravo-Risi et al. 2023). Prions can be exchanged directly between individuals, leading to infection (Haley and Hoover 2015; Grear et al. 2010). They also readily bind to soil, where they may be taken up and sequestered in plant tissues (Pritzkow et al. 2015). Prion-contaminated soil is capable of infecting healthy deer (Mathiason et al. 2009; Nichols et al. 2013) and can remain infectious for years (Otero et al. 2021). Thus soil has been posited as a primary route of infection (Plummer et al. 2018). In the only study of CWD in captive cervid populations, Miller et al. (2006) recorded yearly deaths in herds of penned mule deer (*O. hemionus*) infected with CWD. The first cohort of deer had all died by the end of 1984. For 8 years the pen sat vacant, then a new cohort, believed to be healthy, was introduced in 1992. In a striking demonstration of prions’ persistence in the wild, CWD broke out in this new population, killing the entire cohort by 2001.

Recent evidence suggests that CWD’s indirect transmission pathway exhibits threshold dynamics. Denkers et al. (2020) found that deer inoculated with 300 ${ng}$ of CWD-infected brain (or an equivalent amount of saliva) became infected after 3 weekly doses of 100 ${ng}$, but not after 12 weekly doses of 30 ${ng}$. Thus infection is unlikely to occur unless a sufficient amount of infectious material has been deposited into the environment and deer are exposed to an infectious dose. McClure and Powell (2024) found that threshold transmission led to a strong Allee-like effect in the production of infectives. This behavior generated pushed fronts, which are driven by a combination of host movement and infectious growth. Pushed fronts are qualitatively distinct from pulled fronts, which arise from linear instability of the uninfected steady state, and therefore are less dependent on the specific movement patterns of hosts. These front types motivate different mitigation strategies; pulled fronts are typically addressed by population culling or lowering contact rates to bring the basic reproduction number of a disease below one, while pushed fronts may be better managed by addressing hotspots of prion accumulation or limiting dispersal from infectious areas.

One limitation of the model in McClure and Powell (2024) was its assumption that transmission rates, movement tendencies, etc. were constant across space. In reality, deer movement and behavior is highly correlated with land cover type (Gilbertson et al. 2022). Land cover distribution is highly heterogeneous over the scales of interest for CWD spread; this imbues the CWD study area with a patchy quality that, famously, may produce counterintuitive results relative to those from models of homogeneous landscapes (Musgrave et al. 2015; Duncan et al. 2017; Maciel and Lutscher 2018; Urquhart and Williams 2021). It is computationally prohibitive to simulate a model incorporating small-scale variation; the computational cost of simulating a typical PDE model scales linearly with the number of time steps and often with the *square* of the number of spatial grid points due to stability requirements.

Homogenization is a powerful technique to rectify these issues. Originally devised for finding macroscopic physical properties of composites with fine microstructure (Berlyand and Rybalko 2018), homogenization has also been used in biological and ecological systems with multiple scales (Cobbold et al. 2022; Duncan et al. 2017; Powell and Zimmermann 2004). The technique works by taking a multi-scale model and matching asymptotic expressions that come from scale separation, resulting in a model on large scales that asymptotically approximates the original (Holmes 2013; Marchenko and Khruslov 2006). The spatial scales considered in our model of CWD are those of landscape variation (short, on the order of 30 meters) and domains of interest for tracking CWD spread (long, on the scale of kilometers). The two temporal scales are those of movement (on the scale of hours) and disease progression (on the scale of a year).

The spread of CWD is closely related to movement patterns of deer, since prions themselves exhibit low motility in soil (Jacobson et al. 2010). We will focus on white-tail deer (WTD, *O. virginianus*) in the state of Wisconsin (WI), United States as a case study. The dispersal and migratory patterns of WTD vary considerably across the United States (Jennelle et al. 2022), but in southwestern Wisconsin, adult deer rarely migrate or disperse; the vast majority of annual dispersal is undergone by yearlings (Skuldt et al. 2008). The influence of yearling dispersal on CWD spread has been investigated in McClure and Powell (2024); in this paper we are chiefly interested in homogenizing indirect transmission and examining its affect on traveling fronts, so we model WTD motion as purely diffusive. We use ecological diffusion, an established model of animal movement that accommodates the population effects of differential movement in habitat types (Turchin 1998). Ecological diffusion allows for the aggregation of individuals in certain types of habitat (Figure 2), an important factor in disease modeling due to increased contact rates. This diffusion mechanism has already been used to model CWD spread in deer, as in Garlick et al. (2014) and Hefley et al. (2017), who also homogenize their models.

Existing population models for CWD have included ordinary differential equations (ODE) (Miller et al. 2006; Xu et al. 2022) as well as partial differential equations (PDE) like those mentioned above. These models all consider direct and indirect transmission routes; however, there has not yet been a model that incorporates threshold mechanics in the indirect transmission pathway, nor its likely interactions with movement and congregation. The existing models use mass-action kinetics to model indirect transmission, and therefore may miss crucial dynamics that drive CWD spread. In McClure and Powell (2024), we saw a transition from pushed to pulled fronts as population density increases. Since best mitigation strategies depend on transmission dynamics, it is imperative to understand this behavior in the context of highly variable landscapes. A homogenized model may inform the best strategies for different habitat configurations across disparate landscapes.

In this paper, we construct a multi-scale PDE model of CWD spread that incorporates indirect transmission subject to thresholds. The coefficients related to individual behavior vary on a short spatial scale representing land cover variation. This model is homogenized, yielding a novel nonlinear functional response to threshold transmission on large spatial scales. Using parameters from Miller et al. (2006) and Hefley et al. (2017), we investigate CWD spread behavior in WI. We determine the necessary criteria for pulled and pushed fronts and use numerical simulations to validate front speed estimates. We also use the homogenized model to investigate a virtual landscape with periodic spatial structure and predict outcomes and best intervention strategies in the true small-scale system.

## Model Formulation

To begin, we construct a model of CWD transmission incorporating movement and transmission dynamics on short spatial scales. The model consists of three populations: susceptible individuals, $s({{\textbf {x}}},t)$, infective individuals, $i({{\textbf {x}}},t)$, and environmental prion hazard, $h({{\textbf {x}}},t)$. Specifically, *s*, *i*,  and *h* represent the areal densities of each population at a location ${{\textbf {x}}} = (x_1, x_2) \in {\mathbb {R}}^2$ and time $t\in [0,\infty ).$ The scale of ${{\textbf {x}}}$ is in tens of meters, the resolution of land cover data. Meanwhile *t* is on the order of one hour, a natural timescale for considering the movement of WTD in the WI landscape.

**Fig. 1:**
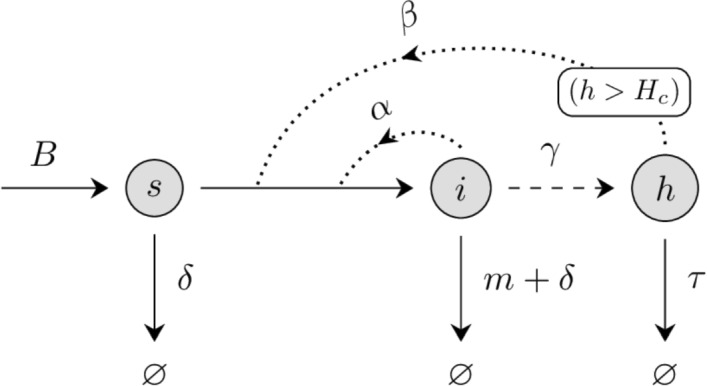
A flow diagram depicting the demographic transitions modeled in (1)

We assume direct contact between susceptibles and infectives causes infection according to mass-action kinetics. Susceptibles may also be indirectly infected through contact with environmental prion, provided local concentration surpasses a threshold. Infective individuals experience CWD mortality without the chance of recovery. An additional natural mortality is applied to both susceptibles and infectives. Deer are born susceptible according to a density-dependent process. Vertical transmission of CWD is known to occur (Nalls et al. 2013), but CWD prevalence in fawns is rare (Grear et al. 2010), so here we assume all newly born deer are susceptible. Finally, environmental prion density increases proportionate to the infectious population, but becomes bio-unavailable over time (Miller et al. 2006). These effects are visualized in a flow diagram in Figure 1, and encapsulated in the following partial differential equations:

1a $$\begin{aligned} \partial _{t}s&= \nabla ^2[\mu ({{\textbf {x}}})s] + \varepsilon ^2\left[ -\alpha si - \beta s(h-H_c)_+ - m s + B \right] ,\end{aligned}$$

1b $$\begin{aligned} \partial _{t}i&= \nabla ^2[\mu ({{\textbf {x}}}) i] + \varepsilon ^2\left[ \alpha si + \beta s(h-H_c)_+ - (m + \delta ) i\right] , \end{aligned}$$

1c $$\begin{aligned} \partial _{t}h&= \varepsilon ^2\left[ \gamma i - \tau h\right] . \end{aligned}$$

The two parameters $\alpha$ and $\beta$ are transmissivities for direct and indirect transmission, respectively. We use the notation $(a)_+ = \text {max}(a,0)$ to refer to the positive part of quantity *a* (Hunter and Nachtergaele 2001); thus environmental prions only infect susceptibles when their concentration exceeds the critical threshold, $H_c$. Natural mortality is given by *m* and CWD mortality by $\delta$. The excretion rate, $\gamma$, describes prion shedding by infectives, and $\tau$ describes the rate at which prion becomes bio-unavailable. Finally, the birth term $B = B(s,i)$ accounts for new arrivals into the susceptible population. A summary of parameter meanings, units, and nominal values is provided in Table 1.

**Fig. 2:**
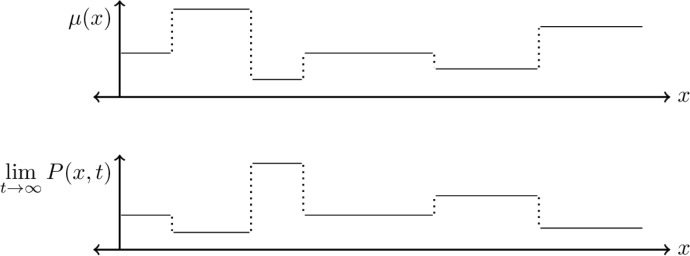
Top: a sample motility function $\mu (x)$. Bottom: the equilibrium solution *P* to the ecological diffusion equation $\partial _{t}P = \nabla ^2[\mu P]$ with the given motility

Of particular importance is the ecological diffusion model for movement, in the form $\nabla ^2[\mu P]$ above. The motility function, $\mu ({{\textbf {x}}})$, encodes individuals’ propensity for movement (motility) in various locales. The higher $\mu$ is at one location, the more likely deer are to move from that location. Thus equilibrium densities of deer will be inversely proportional to the motility at that location in the absence of other factors (Turchin 1998, also see Figure 2). Susceptibles and infectives are assumed to have the same movement behaviors in this model.

Since demographic effects, including disease transmission, are slow relative to small scale movement, we introduce the order parameter $\varepsilon$, $0 < \varepsilon \ll 1$. This represents a separation of time scales between movement – which occurs over hours and days – and the infectious course of CWD – which takes more than a year. The square on $\varepsilon$ in the model equations anticipates parabolic scaling between space and time, a common phenomenon in models with diffusive spread (Holmes 2013).

We also note here that quantities related to prions may be scaled such that the threshold concentration is $H_c = 1$. Let ${\widehat{\beta }} = \beta H_c$, ${\widehat{h}} = h/H_c$, and ${\widehat{\gamma }} = \gamma /H_c$; substitute these parameters into (1), then remove the hats to perform this scaling. This effectively makes one infectious dose the unit of prion concentration, since indirect infection only occurs when $h > 1.$ However, we continue explicitly writing $H_c$ to track units.

## Homogenization

### Introducing Multiple Scales

The model provided in (1) suitably describes changes in local populations over hours, but the scales used are difficult to integrate over kilometers and years. To rectify this, we introduce the long spatial variable, ${{\textbf {X}}}$, and slow time variable, *T*, subject to the relations ${{\textbf {X}}}=\varepsilon {{\textbf {x}}}$ and $T = \varepsilon ^2t$. Thus an *O*(1) change in ${{\textbf {x}}}$ corresponds to a $O(\varepsilon )$ change in ${{\textbf {X}}}$, and an $O(\varepsilon ^2)$ change in *T* to an *O*(1) change in *t* (Powell and Zimmermann 2004). Slow time, *T*, has the natural scale for considering the progress of CWD, while fast time, *t*, is appropriate for motion. Long space, ${{\textbf {X}}}$, has a scale characteristic of deer movement, while the scale of short space, ${{\textbf {x}}}$, is proportional to landscape variation.

The spatially varying motility parameter, $\mu$, may depend on both ${{\textbf {x}}}$ and ${{\textbf {X}}}$. We follow Garlick et al. (2011) and assume $\mu$ is quasi-periodic on the short scale; that is,

2 $$\begin{aligned} \mu ({{\textbf {x}}},{{\textbf {X}}}) = \mu ({{\textbf {x}}} + {{\textbf {p}}}({{\textbf {X}}}),{{\textbf {X}}}) \end{aligned}$$

for some smooth function ${{\textbf {p}}} = (p_1,p_2):{\mathbb {R}}^2 \rightarrow {\mathbb {R}}^2$, which encodes information about landscape structure. Landscapes are obviously not strictly periodic, but they do have repeating features. The quasi-periodicity assumption states that features do repeat on the short scale, but the exact period of repetition depends on location on the long scale. Our analysis heavily leverages periodic boundary conditions on the short scale, so the quasi-periodic assumption is necessary for our analysis. But it is also a realistic assumption; as explained in Garlick et al. (2011), this is equivalent to assuming the Fourier spectrum of $\mu$ is concentrated around one wave number. The quasi-periodic structure motivates the definition of a cell at each ${{\textbf {X}}}$,

3 $$\begin{aligned} \Omega _{{\textbf {X}}} = \{({{\textbf {x}}},{{\textbf {X}}})\, |\, {{\textbf {x}}} \in [0,{{\textbf {p}}}({{\textbf {X}}}))\}. \end{aligned}$$

The three dependent variables *s*, *i* and *h* are assumed to depend on ${{\textbf {x}}}, {{\textbf {X}}}, t$ and *T*. Additionally, we formally expand each variable as a power series in $\varepsilon$:

4 $$\begin{aligned} s = s_0({{\textbf {x}}},{{\textbf {X}}},t,T) + \varepsilon s_1({{\textbf {x}}},{{\textbf {X}}},t,T) + \varepsilon ^2s_2({{\textbf {x}}},{{\textbf {X}}},t,T) + \ldots , \end{aligned}$$

and similarly for *i* and *h*. Finally, the introduction of multiple scales causes the differential operators in (1) to transform according to the multivariate chain rule:

5a $$\begin{aligned} \partial _{t}&\mapsto \partial _{t} + \varepsilon ^2\partial _{T},\end{aligned}$$

5b $$\begin{aligned} \nabla&\mapsto \nabla _{{\textbf {x}}} + \varepsilon \nabla _{{\textbf {X}}}, \end{aligned}$$

where  and .

With these changes in place, the PDE (1) becomes

6a $$\begin{aligned}&\begin{aligned}&(\partial _{t}+\varepsilon ^2\partial _{T})(s_0 + \varepsilon s_1 + \varepsilon ^2s_2 + \ldots )\\&\quad = (\nabla _{{\textbf {x}}} + \varepsilon \nabla _{{\textbf {X}}})^2[\mu (s_0 + \varepsilon s_1 + \varepsilon ^2s_2 + \ldots )]\\&\qquad - \varepsilon ^2[\alpha s_0i_0 + \beta s_0(h_0-H_c)_+ + B(s_0,i_0)] + O(\varepsilon ^3), \end{aligned} \end{aligned}$$

6b $$\begin{aligned}&\begin{aligned}&(\partial _{t}+\varepsilon ^2\partial _{T})(i_0 + \varepsilon i_1 + \varepsilon ^2i_2 + \ldots )\\&\quad = (\nabla _{{\textbf {x}}} + \varepsilon \nabla _{{\textbf {X}}})^2[\mu (i_0 + \varepsilon i_1 + \varepsilon ^2i_2 + \ldots )]\\&\qquad + \varepsilon ^2[\alpha s_0i_0 + \beta s_0(h_0-H_c)_+ - \delta i_0] + O(\varepsilon ^3), \end{aligned} \end{aligned}$$

6c $$\begin{aligned}&(\partial _{t}+\varepsilon ^2\partial _{T})(h_0 + \varepsilon h_1 + \varepsilon ^2h_2 + \ldots )\nonumber \\&\quad = \varepsilon ^2[\gamma i_0 - \tau h_0] + O(\varepsilon ^3). \end{aligned}$$

Initial conditions for *s*, *i*,  and *h* are assumed to be *O*(1). Boundary conditions will be discussed in the following subsection.

### Balancing and Homogenized Equations

The lowest-order terms to balance are *O*(1); from (6),

7a $$\begin{aligned} \partial _{t} s_0&= \nabla _{{\textbf {x}}}^2[\mu s_0],\end{aligned}$$

7b $$\begin{aligned} \partial _{t} i_0&= \nabla _{{\textbf {x}}}^2[\mu i_0],\end{aligned}$$

7c $$\begin{aligned} \partial _{t} h_0&= 0. \end{aligned}$$

Equation (7c) implies $h_0$ is constant on small time scales, $h_0 = h_0({{\textbf {x}}},{{\textbf {X}}},T).$ Equations (7ab) are parabolic, so transients in *t* decay exponentially and may be disregarded in the long *t* limit. Seeking steady-state solutions in *t*, we drop *t* from the arguments of $s_0, i_0,$ and $h_0$, leaving

8 $$\begin{aligned} 0 = \nabla _{{\textbf {x}}}^2[\mu s_0] \quad \text{ and }\quad 0 = \nabla _{{\textbf {x}}}^2[\mu i_0]. \end{aligned}$$

These PDEs are Laplace’s equation in ${{\textbf {x}}}$ for the quantities $\mu s_0$ and $\mu i_0$. Consider this equation on the cell $\Omega _{{\textbf {X}}}$ defined in (3). To prevent secularity of $s_0$ and $i_0$ in ${{\textbf {x}}}$, we impose a periodic boundary condition for $\mu s$, $\mu i$ and their gradients on opposing points of the cell. The solution to (8) with these boundary conditions has $\mu s_0$ and $\mu i_0$ constant with respect to ${{\textbf {x}}}$:

9 $$\begin{aligned} \mu s_0 = \overline{\mu }S({{\textbf {X}}},T) \quad \text{ and }\quad \mu i_0 = \overline{\mu }I({{\textbf {X}}},T). \end{aligned}$$

Here we introduce the quantity $\overline{\mu }$ to balance units. If $\overline{\mu }$ is chosen to be the harmonic mean of $\mu$ on the cell,

10 $$\begin{aligned} \overline{\mu }({{\textbf {X}}})^{-1} = \frac{1}{|\Omega _{{\textbf {X}}}|}\iint _{\Omega _{{\textbf {X}}}} \frac{1}{\mu ({{\textbf {x}}},{{\textbf {X}}})}\,\text {d}{{\textbf {x}}} \end{aligned}$$

then $\overline{\mu }S$ and $\overline{\mu }I$ may be interpreted as local averages of $\mu s_0$ and $\mu i_0$ at a given ${{\textbf {X}}}$. Thus, the first-order approximations to *s* and *i* satisfy

11 $$\begin{aligned} s_0({{\textbf {x}}},{{\textbf {X}}},T) = \frac{\overline{\mu }}{\mu ({{\textbf {x}}})}S({{\textbf {X}}},T) \quad \text{ and }\quad i_0({{\textbf {x}}},{{\textbf {X}}},T) = \frac{\overline{\mu }}{\mu ({{\textbf {x}}})}I({{\textbf {X}}},T). \end{aligned}$$

This conclusion is ecologically sound, essentially stating that susceptibles and infectives are in approximate equilibrium with their habitat preference.

The next order to balance is $O(\varepsilon )$. Collecting these terms from (6) yields

12a $$\begin{aligned} \partial _{t} s_1&= \nabla _{{\textbf {x}}}^2[\mu s_1] + 2\nabla _{{\textbf {x}}}\cdot \nabla _{{\textbf {X}}}[\mu s_0], \end{aligned}$$

12b $$\begin{aligned} \partial _{t} i_1&= \nabla _{{\textbf {x}}}^2[\mu i_1] + 2\nabla _{{\textbf {x}}}\cdot \nabla _{{\textbf {X}}}[\mu i_0], \end{aligned}$$

12c $$\begin{aligned} \partial _{t} h_1&= 0. \end{aligned}$$

From (9), the products $\mu s_0$ and $\mu i_0$ have no dependence on ${{\textbf {x}}}$, and therefore the mixed partial derivative terms vanish. As before, we seek equilibrium solutions in *t*, leaving

13 $$\begin{aligned} 0 = \nabla _{{\textbf {x}}}^2[\mu s_1] \quad \text{ and }\quad 0 = \nabla _{{\textbf {x}}}^2[\mu i_1]. \end{aligned}$$

This is the same problem as (8), so $s_1$ and $i_1$ have the same form as in (11). Initial conditions for *s* and *i* are *O*(1) only, so $s_1$ and $i_1$ provide no correction to the *O*(1) solution given by $s_0$ and $i_0$. Thus we may safely choose $s_1 = i_1 \equiv 0$ and move on. Additionally, the *O*(1) initial conditions given to *h* imply $h_1(t=0) = 0$, which combined with (12c) gives $h_1 \equiv 0$.

When collecting $O(\varepsilon ^2)$ terms from (6), the reaction terms appear:

14a $$\begin{aligned} \partial _{T}s_0 + \partial _{t}s_2&= \nabla _{{\textbf {x}}}^2[\mu s_2] + 2\nabla _{{\textbf {x}}}\cdot \nabla _{{\textbf {X}}}[\mu s_1] + \nabla _{{\textbf {X}}}^2[\mu s_0] \nonumber \\&\qquad - \alpha s_0 i_0 - \beta s_0(h_0-H_c)_+ -ms_0 + B,\end{aligned}$$

14b $$\begin{aligned} \partial _{T}i_0 + \partial _{t}i_2&= \nabla _{{\textbf {x}}}^2[\mu i_2] + 2\nabla _{{\textbf {x}}}\cdot \nabla _{{\textbf {X}}}[\mu i_1] + \nabla _{{\textbf {X}}}^2[\mu i_0] \nonumber \\&\qquad + \alpha s_0 i_0 + \beta s_0(h_0-H_c)_+ - (m + \delta ) i_0,\end{aligned}$$

14c $$\begin{aligned} \partial _{T}{h_{0}} + \partial _{t}{h}_{2}&= \gamma {i}_{0} -\tau {h}_{0}. \end{aligned}$$

Once again derivative terms like $\partial _{t}s_2$ are set to zero in the pursuit of equilibrium solutions on fast time scales. Since $s_1$ and $i_1$ are zero, neglecting transients in *t*, and using (11), equations (14) may be rewritten

15a $$\begin{aligned} \nabla _{{\textbf {x}}}^2[\mu s_2]&= \frac{\overline{\mu }}{\mu }\partial _{T}S - \overline{\mu }\nabla _{{\textbf {X}}}^2S + \alpha \frac{\overline{\mu }^2}{\mu ^2}SI + \beta \frac{\overline{\mu }}{\mu }S(h_0-H_c)_+ + m\frac{\overline{\mu }}{\mu }S - B, \end{aligned}$$

15b $$\begin{aligned} \nabla _{{\textbf {x}}}^2[\mu i_2]&= \frac{\overline{\mu }}{\mu }\partial _{T}I - \overline{\mu }\nabla _{{\textbf {X}}}^2I - \alpha \frac{\overline{\mu }^2}{\mu ^2}SI - \beta \frac{\overline{\mu }}{\mu }S(h_0-H_c)_+ + (m+\delta )\frac{\overline{\mu }}{\mu }I, \end{aligned}$$

15c $$\begin{aligned} \partial _{T}h_0&= \gamma \frac{\overline{\mu }}{\mu }I - \tau h_0. \end{aligned}$$

Consider the equilibrium distribution of $h_0$ in *T*: from (15c),

16 $$\begin{aligned} h_0 = \frac{\gamma }{\tau } \frac{\overline{\mu }}{\mu ({{\textbf {x}}})} I. \end{aligned}$$

This motivates the change of variables

17 $$\begin{aligned} h_0({{\textbf {x}}},{{\textbf {X}}},T) = \frac{\overline{\mu }}{\mu ({{\textbf {x}}})}H({{\textbf {x}}},{{\textbf {X}}},T). \end{aligned}$$

Substituting this form into (15c) yields

18 $$\begin{aligned} \partial _{T} H = \gamma I - \tau H. \end{aligned}$$

Assuming initial conditions are of the form $h_0({{\textbf {x}}},{{\textbf {X}}},0) = \frac{1}{\mu ({{\textbf {x}}})}f({{\textbf {X}}})$, (18) suggests that *H* may be taken not to depend on ${{\textbf {x}}}$, so $H = H({{\textbf {X}}},T)$. Our system of PDEs at $O(\varepsilon ^2)$ then finally simplifies to

19a $$\begin{aligned} \nabla _{{\textbf {x}}}^2[\mu s_2]&= \frac{\overline{\mu }}{\mu }\partial _{T}S - \overline{\mu }\nabla _{{\textbf {X}}}^2S + \alpha \frac{\overline{\mu }^2}{\mu ^2}SI + \beta \frac{\overline{\mu }}{\mu }S\left( \frac{\overline{\mu }}{\mu }H-H_c\right) _+ + m\frac{\overline{\mu }}{\mu }S - B, \end{aligned}$$

19b $$\begin{aligned} \nabla _{{\textbf {x}}}^2[\mu i_2]&= \frac{\overline{\mu }}{\mu }\partial _{T}I - \overline{\mu }\nabla _{{\textbf {X}}}^2I - \alpha \frac{\overline{\mu }^2}{\mu ^2}SI - \beta \frac{\overline{\mu }}{\mu }S\left( \frac{\overline{\mu }}{\mu } H-H_c\right) _+ + (m+\delta )\frac{\overline{\mu }}{\mu }I, \end{aligned}$$

19c $$\begin{aligned} \partial _{T}H&= \gamma I - \tau H. \end{aligned}$$

For now let us restrict our attention to (19a). To continue the expansion we would need to solve for $s_2$; the Fredholm alternative theorem provides conditions under which a solution exists. Let $C^2(\Omega _{{\textbf {X}}})$ denote the space of functions on $\Omega _{{\textbf {X}}}$ twice differentiable with respect to ${{\textbf {x}}}$ and satisfying the same periodic boundary conditions as *s*, *i* and *h*. This is a Hilbert space when equipped with the inner product

20 $$\begin{aligned} f\cdot g = \frac{1}{|\Omega _{{\textbf {X}}}|}\iint \limits _{\Omega _{{\textbf {X}}}} fg\,\text {d}{{\textbf {x}}} = \frac{1}{p_1({{\textbf {X}}})p_2({{\textbf {X}}})}\int _{0}^{p_2({{\textbf {X}}})}\int _{0}^{p_1({{\textbf {X}}})} fg\,\text {d}x_1\text {d}x_2. \end{aligned}$$

We consider $s_2$ as an element of $C^2(\Omega _{{\textbf {X}}})$, ignoring its dependency on ${{\textbf {X}}}$ and *T*. Now let *L* be the differential operator on $C^2(\Omega _{{\textbf {X}}})$ defined by

21 $$\begin{aligned} Lu = \nabla _{{\textbf {x}}}^2[\mu u]. \end{aligned}$$

Its adjoint in $C^2(\Omega _{{\textbf {X}}})$ is given by $L^*$ satisfying

22 $$\begin{aligned} L^*u = \mu \nabla _{{\textbf {x}}}^2u. \end{aligned}$$

As a consequence of the Fredholm Alternative Theorem, (19a) has a unique solution $s_2$ if and only if for any $u \in \ker (L^*)$ we have $u \cdot \text {RHS} = 0$, where $\text {RHS}$ is the right-hand side of (19a). Suppose then that $u \in \ker (L^*)$. That is,

23 $$\begin{aligned} L^*u = \mu \nabla _{{\textbf {x}}}^2u = 0, \quad \text{ or }\quad \nabla _{{\textbf {x}}}^2u = 0. \end{aligned}$$

This is again Laplace’s equation subject to periodic boundary conditions, so *u* is constant with respect to ${{\textbf {x}}}$, say $u = k$. To ensure a unique solution to (19a), it must be the case that $u\cdot \text {RHS} = 0,$ or

24 $$\begin{aligned} \frac{1}{|\Omega _{{\textbf {X}}}|}\iint \limits _{\Omega _{{\textbf {X}}}} k\left( \frac{\overline{\mu }}{\mu }\partial _{T}S - \overline{\mu }\nabla _{{\textbf {X}}}^2S + \alpha \frac{\overline{\mu }^2}{\mu ^2}SI + \beta \frac{\overline{\mu }}{\mu }S\left( \frac{\overline{\mu }}{\mu }H-H_c\right) _+ + m\frac{\overline{\mu }}{\mu }S - B\right) \,\text {d}{{\textbf {x}}} = 0. \end{aligned}$$

We rewrite this equation as

25 $$\begin{aligned} &  \overline{\mu }\left<\frac{1}{\mu }\right>\partial _{T}S - \overline{\mu }\nabla _{{\textbf {X}}}^2S + \overline{\mu }^2\left<\frac{\alpha }{\mu ^2}\right>SI + \beta S\frac{1}{|\Omega _{{\textbf {X}}}|}\iint \limits _{\Omega _{{\textbf {X}}}}\frac{\overline{\mu }}{\mu }\left( \frac{\overline{\mu }}{\mu }H - H_c \right) _+\text {d}{{\textbf {x}}}\nonumber \\ &  \qquad + m\overline{\mu }\left<\frac{1}{\mu } \right>S - \left<B \right>= 0, \end{aligned}$$

where we define $\left<f \right>$ as the average of *f* across a cell:

26 $$\begin{aligned} \left<f({{\textbf {x}}},{{\textbf {X}}})\right> = \frac{1}{|\Omega _{{\textbf {X}}}|} \iint \limits _{\Omega _{{\textbf {X}}}} f({{\textbf {x}}},{{\textbf {X}}})\,\text {d}{{\textbf {x}}}. \end{aligned}$$

Using this notation the homogenized motility may be expressed $\overline{\mu } = \left<1 /\mu \right>^{-1}$ (10). Equation (25) then becomes

27 $$\begin{aligned} \partial _{T}S = \overline{\mu } \nabla _{{\textbf {X}}}^2S - \overline{\alpha } SI - \beta SF(H) - mS + \left<B \right>, \end{aligned}$$

where $\overline{\alpha }$ is the homogenized direct transmissivity,

28 $$\begin{aligned} \overline{\alpha } = \overline{\mu }^2\left<\frac{\alpha }{\mu ^2}\right>, \end{aligned}$$

and *F* is a function satisfying

29 $$\begin{aligned} F(H) = \frac{1}{|\Omega _{{\textbf {X}}}|} \iint \limits _{\Omega _{{\textbf {X}}}} \frac{\overline{\mu }}{\mu } \left( \frac{\overline{\mu }}{\mu } H - H_c \right) _+\,\text {d}{{\textbf {x}}} = \frac{1}{|\Omega _{{\textbf {X}}}|}\iint _{\Omega _{{\textbf {X}}}} \frac{\overline{\mu }^2}{\mu ^2} \left( H - \frac{\mu }{\overline{\mu }}H_c \right) _+\text {d}{{\textbf {x}}}.\nonumber \\ \end{aligned}$$

A similar argument may be applied to (19b), leading to the following system of homogenized PDEs:

30a $$\begin{aligned} \partial _{T}S&= \overline{\mu }\nabla ^2S - \overline{\alpha }SI - \beta SF(H) - mS + \left<B \right>, \end{aligned}$$

30b $$\begin{aligned} \partial _{T}I&= \overline{\mu }\nabla ^2I + \overline{\alpha }SI + \beta SF(H) - (m+\delta ) I, \end{aligned}$$

30c $$\begin{aligned} \partial _{T}H&= \gamma I - \tau H. \end{aligned}$$

Equations (30) superficially resemble the small-scale PDE system (1) with important, computationally beneficial differences. Homogenization takes the motility function out from inside the Laplacian in ecological diffusion terms. The mass-action terms – direct transmission, infectious death, hazard deposition and decay – homogenize up to mass-action terms as well. Indirect transmission, by contrast, depends on *H* nonlinearly; its expression through the *F* function in (30) requires independent evaluation.

Since (30) depends only on ${{\textbf {X}}}$ and *T*, the spatial resolution changes from $\varepsilon$ to 1 and the temporal resolution from $\varepsilon ^2$ to 1, in comparison to (1). This reduces the computation time of numerically integrating the PDE by a factor of $\varepsilon ^4$ when using an explicit numerical scheme (Garlick et al. 2011). In an ideal scenario, the error incurred by using (30) to approximate solutions to (1) would be $O(\varepsilon ^2)$, since over the course of homogenization we balance terms up to $O(\varepsilon )$. However, this assumes that integrals of the form $\left<f \right>$ can be exactly computed. In reality, the quasi-period ${{\textbf {p}}}$ functions is not known and integrations to approximate $\left<f \right>$ must accommodate. This introduces a bottleneck error of $O(\varepsilon ^p)$, where $0<p<1$; For more details, refer to Appendix A.

### Effects of Indirect Transmission on Large Scales

It remains to explicitly characterize the indirect transmission term in (30). We assume motility is piecewise constant in space,31since land cover data is usually provided as a raster (Azubike et al. 2022). Here $\{\mu _i\}_{i\in \{1,\ldots ,n\} }$ are discrete values ordered so $\mu _1< \ldots < \mu _n$; $\{S_i\}_{i\in \{1,\ldots ,n\}}$ are mutually disjoint sets such that $\cup _{i=1}^n S_i = \Omega _{{\textbf {X}}}$; and  denotes an indicator function. To evaluate the integral defining *F* in (29), it is helpful to apply an ergodic assumption on the motility function $\mu$. We assume that $\mu _i$ are samples from a probability density function, $\rho (\mu )$. The probability mass, $p_i$, for each $\mu _i$ is then

32 $$\begin{aligned} p_i \approx \rho (\mu _i)\Delta \mu _i, \end{aligned}$$

where $\Delta \mu _i = \mu _{i+1} - \mu _i$. From another perspective, $p_i$ can be written as the proportion of $\Omega _{{\textbf {X}}}$ supporting $\mu _i$, so that $p_i = |S_i|/|\Omega _{{\textbf {X}}}|$.

Substituting (31) into (29) yields

33 $$\begin{aligned} F(H) = \frac{1}{|\Omega _{{\textbf {X}}}|}\sum _{i=1}^{n} \iint _{S_i} \frac{\overline{\mu }^2}{\mu _i^2}\left( H - H_i \right) _+\text {d}{{\textbf {x}}} \end{aligned}$$

where

34 $$\begin{aligned} H_i = \frac{\mu _i}{\overline{\mu }}H_c. \end{aligned}$$

Let $$F_i = F(H_i)$$. What follows is a computation of *F*(*H*) on each interval $$[H_i,H_{i+1})$$, leading to the derivation of successive $$F_i$$ values. After three successive cases a recurrence relation appears, leading to a differential equation that may be solved for *F*.

**Fig. 3:**
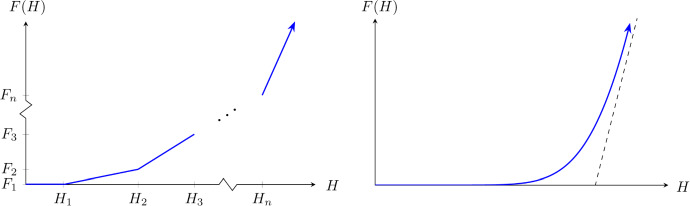
Left: the graph of *F* when $$\mu $$ is piecewise constant. Note that the slope of *F* increases in each successive interval $$[H_{i-1},H_i)$$. Right: the graph of *F* when $$\mu $$ is a continuous function with a gamma distribution (Color figure online)

#### Case 1: $H \in [0,H_1)$

In this case $(H - H_i)_+ = 0$ for all *i*, so $F(H) = 0$ over this interval and $F_1 = 0.$

#### Case 2: $H\in [H_1,H_2)$

In this case (33) simplifies to

35 $$\begin{aligned} F(H) = \frac{1}{|\Omega _{{\textbf {X}}}|}\iint _{S_1} \frac{\overline{\mu }^2}{\mu _1^2} (H - H_1)\,\text {d}{{\textbf {x}}} = \frac{|S_1|}{|\Omega _{{\textbf {X}}}|} \frac{\overline{\mu }^2}{\mu _1^2}(H - H_1) = p_1\frac{\overline{\mu }^2}{\mu _1^2} (H-H_1).\nonumber \\ \end{aligned}$$

Thus on the interval $$[H_1,H_2)$$, *F* is a line with slope $$m_1 = p_1 \dfrac{\overline{\mu }^2}{\mu _1^2}$$. We may say $$F_2 = m_1\Delta H_1$$, where $$\Delta H_i = H_{i+1}-H_i.$$

#### Case 3: $H \in [H_2,H_3)$

By similar reasoning, (33) in this case becomes

36 $$\begin{aligned} F(H) = p_1\frac{\overline{\mu }^2}{\mu _1^2}(H-H_1) + p_2\frac{\overline{\mu }^2}{\mu _2^2}(H-H_2), \end{aligned}$$

so that *F* is a line with slope $m_2 = m_1 + p_2\dfrac{\overline{\mu }^2}{\mu _2^2}$. We may say $F_3 = F_2 + m_2\Delta H_2$.

#### The General Case

The pattern established from these cases continues until $H > H_n$. In general, we have the recurrence relations

37a $$\begin{aligned} F_{i+1}&= F_i + m_i\Delta H_i, \end{aligned}$$

37b $$\begin{aligned} m_i&= m_{i-1} + p_i\frac{\overline{\mu }^2}{\mu _i^2}. \end{aligned}$$

Introducing the probability density from (32) into (37b) yields

38 $$\begin{aligned} m_i \approx m_{i-1} + \rho (\mu _i)\frac{\overline{\mu }^2}{\mu _i^2}\Delta \mu _i \end{aligned}$$

and (34) allows this equation to be expressed in terms of $\Delta H_i$, allowing comparison with (37a):

39 $$\begin{aligned} m_i \approx m_{i-1} + \rho \left( \overline{\mu }\frac{H_i}{H_c}\right) \frac{\overline{\mu }H_c}{H_i^2}\Delta H_i. \end{aligned}$$

Now (37a) and (39) may be rearranged

40a $$\begin{aligned} \frac{\Delta F_i}{\Delta H_i}&= m_i,\end{aligned}$$

40b $$\begin{aligned} \frac{\Delta m_{i-1}}{\Delta H_i}&\approx \rho \left( \overline{\mu }\frac{H_i}{H_c}\right) \frac{\overline{\mu }H_c}{H_i^2}. \end{aligned}$$

In the limit $\Delta H_i \rightarrow 0$, 41a41b or more simply42subject to the initial conditions $F(0) = F'(0) = 0$ (which follow from Case 1 above).

When a density function $\rho$ is provided, (42) may be solved explicitly for *F*. To prevent singular behavior, the density should satisfy $\rho (\mu ) = O(\mu ^2)$ as $\mu \rightarrow 0$. Equivalently, $\rho (0) = \rho '(0) = 0.$ Ecologically speaking, these conditions forbid locations where motility approaches zero and corresponding population densities could become unbounded. One distribution satisfying these requirements is the gamma distribution with shape parameter equal to 3,

43 $$\begin{aligned} \rho (\mu ) = \frac{1}{2}\lambda ^3\mu ^2e^{-\lambda \mu }, \end{aligned}$$

where $\lambda$ is a scale parameter. This distribution agrees well with observed GPS collar data from Wisconsin WTD, so we will use it in our most plausible model of CWD in subsection 5.2.

This particular form of $\rho$ allows us to explicitly compute homogenized quantities. For example,

$$\begin{aligned} \overline{\mu }^{-1} = \frac{1}{|\Omega _{{\textbf {X}}}|}\iint _{\Omega _{{\textbf {X}}}}\frac{1}{\mu }\,\text {d}{{\textbf {x}}} \approx \frac{1}{|\Omega _{{\textbf {X}}}|}|\Omega _{{\textbf {X}}}|\ {\mathbb {E}}\left[ \frac{1}{\mu } \right] = \int _{0}^{\infty } \frac{1}{\mu }\rho (\mu ) \,\text {d}\mu = \frac{\lambda }{2} \end{aligned}$$

So that $\overline{\mu } = 2/\lambda$. A similar calculation shows $\overline{\alpha } = 2\alpha$. Additionally, the ODE for *F* provided in (42) can now be solved with the given initial conditions:

44 $$\begin{aligned} F(H) = 2H + H_c\left( e^{-2H/H_c}-1\right) . \end{aligned}$$

A plot of this particular version of *F* can be seen in Figure 3.

## Invasion Speed Analysis for the Homogenized Model

With the homogenized model (30) fully described, we are now prepared to analyze the conditions under which fronts of CWD infection spread, as well as the asymptotic speeds these fronts achieve. The model is first reduced to a univariate PDE for infectives to aid visualization and analytical presentation. We identify necessary conditions for pulled and pushed fronts separately, then discuss the outcome when fronts of both types are possible.

Pulled fronts arise from the linear instability of the uninfected equilibrium, $I = 0$ (Ebert and van Saarloos 2000; Van Saarloos 2003). Their propagation is fed at the leading edge of invasion where infectious growth is greatest (Weinberger 1982). By contrast, pushed fronts do not require the uninfected equilibrium to be unstable. A combination of movement and dynamics throughout the bulk of the front allows a pushed front to advance into uninvaded spaces, overcoming the stability of $I = 0$ (Van Saarloos 2003). Pushed fronts are famously bistable, able to advance in either direction, but here we limit our attention to fronts that spread infective populations rather than shrink them. We thus assume the speed is positive for both pulled and pushed fronts.

### Model Reduction and Fixed Point Analysis

Our homogenized PDE system reduces to two equations if the total population of deer is held constant. We assume that susceptibles are born to replace dying susceptibles and infectives from a long-scale perspective. The replacement of dead susceptibles is expected in a population that has reached its carrying capacity, and the replacement of infectives may arise from compensatory dynamics (Kistner and Belovsky 2014). Therefore $\left<B \right> = mS + (m+\delta ) I$, leaving the total host population at a constant $P = S + I$. Equations (30) then reduce to

45a $$\begin{aligned} \partial _{T} I&= \overline{\mu }\nabla ^2I + \overline{\alpha }(P-I)I + \beta (P-I)F(H) - (m+\delta ) I, \end{aligned}$$

45b $$\begin{aligned} \partial _{T} H&= \gamma I - \tau H. \end{aligned}$$

We also assume quasi-equilibrium between infective density and prion concentration. That is, prion concentration quickly equilibrates to the arrival or departure of infectives. Notationally this is expressed $\partial _{T}H = 0,$ or $H = \frac{\gamma }{\tau }I$, allowing for another reduction:

46 $$\begin{aligned} \partial _{T} I = \overline{\mu }\nabla ^2I + \underbrace{\overline{\alpha }(P-I)I + \beta (P-I)F\left( \frac{\gamma }{\tau } I\right) - (m+\delta ) I}_{:= R(I)}. \end{aligned}$$

Equation (46) is a reaction-diffusion equation with a reaction term *R*(*I*). Note that $R'(0) = \overline{\alpha }P - m - \delta$ since $F'(0) = 0$; this is the classic basic reproduction rate for direct infection. We now restrict our attention to the direction of CWD propagation, and take the scalar spatial variable *X* to be normal to the two-dimensional wave of invasion. This replaces the $I({{\textbf {X}}},T)$ with *I*(*X*, *T*) and $\nabla ^2$ with $\partial _{X}^2$ in equation (46).

**Fig. 4:**
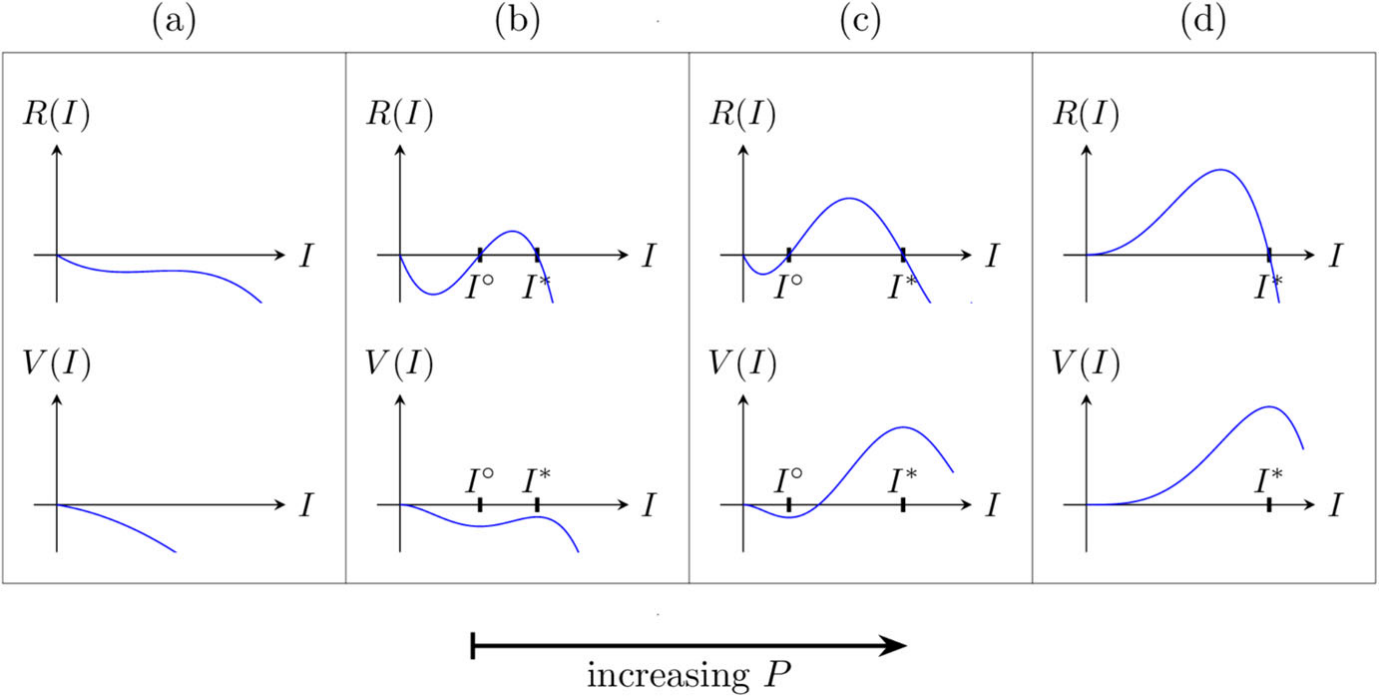
Representative plots of the reaction function, *R*, defined in (46), as well as the potential function $V(I) = \int _{0}^{I} R(y)\,\text {d}y$, for a variety of population densities, *P*. Top plots are *R* and bottom plots are *V*. (a): Population density is too low for any positive equilibria to exist. (b): The positive equilibria $I^\circ$ and $I^*$ exist, but $V(I^*) < 0$ so no fronts develop. The uninfected state is still stable. (c): Population density is large enough that $V(I^*) > 0$, allowing for pushed front behavior. (d): The case where $P > (m+\delta )/\overline{\alpha }$, so the uninfected state is unstable and pulled fronts occur

We begin by analyzing the existence and stability of steady-state solutions to the ODE $\partial _{T}I = R(I)$. Infectious densities that are stable in the absence of movement are exactly the densities that can be connected across space by a traveling front. Equilibrium densities are roots of the reaction function *R*(*I*), which bifurcate with respect to the population density, *P* (Figure 4). For sufficiently low *P* the only equilibrium is the uninfected steady-state, $I = 0$, which is stable. As *P* increases, two positive roots of *R* appear; we label the lesser of these roots $I^\circ$ and the greater one $I^*$. The middle equilibrium $I^\circ$ is unstable; considering the growth of infectives from zero, $I^\circ$ may be thought of as a threshold infective density to exceed before infection can progress to the stable endemic state $I^*$. Thus, in this regime of *P* values, infectious growth is subject to strong Allee-like effects. This situation persists until $P > (m + \delta ) / \overline{\alpha }$, at which point $I = 0$ becomes unstable since $R'(0)>0$, and $I^\circ$ becomes negative and is therefore disregarded.

### Pulled Fronts

Seeking pulled fronts, we linearize (46) around $I = 0$, assumed to be unstable:

47 $$\begin{aligned} \partial _{T}I = \overline{\mu }\partial _{X}^2I + R'(0)I. \end{aligned}$$

We adopt a Galilean frame of reference moving at a constant positive speed *c* with the variable $Z = X - cT$. Then $\partial _{X}\mapsto \partial _{Z},$ $\partial _{T}\mapsto \partial _{T} -c\partial _{Z}$, and changing to $I = I(Z,T)$ yields the following PDE:

48 $$\begin{aligned} \partial _{T}I - c\partial _{Z}I = \overline{\mu }\partial _{Z}^2I + R'(0)I. \end{aligned}$$

At leading order, the linear instability of $I = 0$ will cause exponential growth in *T* and *Z* near 0. Assuming

49 $$\begin{aligned} I \sim e^{\sigma T + \nu Z} \end{aligned}$$

yields the dispersion relation

50 $$\begin{aligned} \sigma = \overline{\mu }\nu ^2 + c\nu + R'(0). \end{aligned}$$

The ansatz (49) describes a continuum of traveling front solutions to (48). As *T* increases, the most observable (asymptotically dominant) solution will be the one with maximum growth rate $\sigma$. The growth rate is maximized when

51 $$\begin{aligned} \partial _{\nu }\sigma = 0 \quad \Rightarrow \quad \nu = -\frac{c}{2\overline{\mu }}, \end{aligned}$$

which, substituted into (50), yields

52 $$\begin{aligned} \sigma = -\frac{c^2}{4\overline{\mu }} + R'(0). \end{aligned}$$

We have just chosen the fastest-growing front solution; now to find the front’s speed, we must chose a reference frame such that the front appears not to grow at all (“surfing the front,” see Duncan et al. 2017). Such a reference frame has $\sigma = 0$; solving (52) for *c* in this case yields

53 $$\begin{aligned} c^* = 2\sqrt{\overline{\mu }R'(0)} = 2\sqrt{\overline{\mu }(\overline{\alpha }P-m-\delta )}. \end{aligned}$$

We adopt the notation $c^*$ to refer to the privileged front speed most observable among pulled fronts. Since $c^*$ does not depend on any terms related to indirect transmission, we may identify pulled front behavior with direct transmission alone.

### Pushed Fronts

For this section assume $I = 0$ is stable. In the Galilean frame of reference, fronts are solutions to the ODE

54 $$\begin{aligned} \overline{\mu }I'' + cI' + R(I) = 0, \end{aligned}$$

where $I = I(Z)$. Multiplying both sides by $I'$ and rearranging:55where $V(I) = \int _{0}^{I} R(u)\,\text {d}u.$ Sample graphs of *V* can be found in Figure 4; the extrema of *V* naturally correspond to equilibria. Equation (55) reads as an energy expression in which the total energy, $\dfrac{1}{2}\overline{\mu }(I')^2 + V(I)$, always decreases in *Z* when $c > 0$. From this perspective *V*(*I*) acts as the system’s potential energy, and *c* becomes a damping constant.

**Fig. 5:**
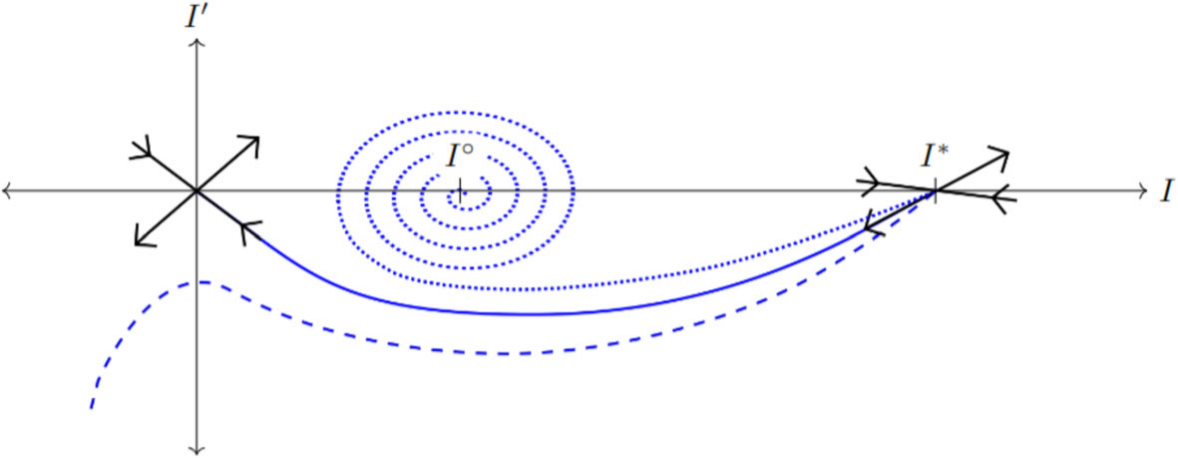
A phase portrait for the ODE (54) with varying values of the front speed, *c*. Black arrows show stable and unstable eigendirections for the fixed points 0 and $I^*$. Blue curves show solutions for (54) starting near the unstable eigendirection of $I^*$. The dashed line is a trajectory when $c < c^\dagger$, the dotted line when $c > c^\dagger$, and the solid line when $c = c^\dagger .$ (Color figure online)

Suppose that parameters are such that $I^\circ$ and $I^*$ both exist. Since the variable *Z* reverses the sign of time, the stable states $I = 0$ and $I^*$ are unstable in the ODE (54); in fact, both are saddle nodes. If $V(I^*) > V(0)$, then there is a unique $c = c^\dagger$ connecting the unstable eigendirection of $I^*$ to the stable eigendirection of $I = 0$ (Powell 1997) (see Figure 5). Values of *c* less than $c^\dagger$ fail to connect these two directions, and values above $c^\dagger$ connect $I^*$ with $I^\circ$ instead. This is analogous to a ball rolling down the graph of *V* from $I^*$ to $I = 0$ (Figure 4); $c^\dagger$ is the unique friction (damping) applied such that the ball stops exactly at $I = 0$ rather than over- or under-shooting.

**Fig. 6:**
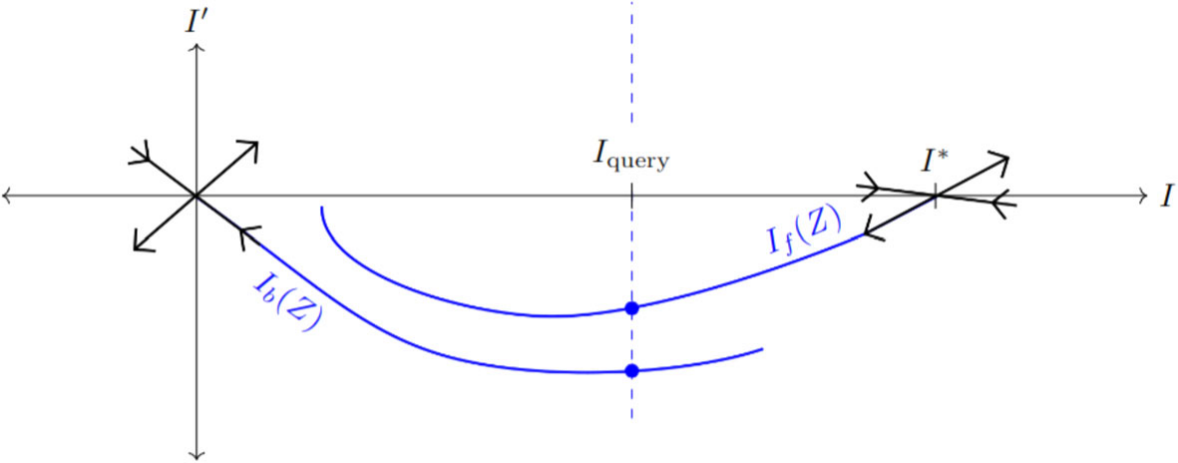
An illustration of the shooting method for determining the pushed front speed $c^\dagger$. Shown is a phase portrait for the ODE (54) for some fixed $c \ne c^\dagger$. A backwards solution $I_b$ is computed from the stable eigendirection of 0, and a forwards solution $I_f$ from the unstable direction of $I^*$. The difference between these solutions at the query value $I_\text {query}$ is minimized to find $c^\dagger$ (Color figure online)

Practically, the value of $c^\dagger$ may be found by numerically shooting trajectories of solutions to (54). The forward solution is computed for an initial value near $I = I^*$ in the unstable eigendirection; call this solution $I_f(Z)$. A backward solution is likewise computed from a point near $I = 0$ in the stable direction, yielding $I_b(Z)$. These trajectories are the same when $c = c^\dagger$, so their difference can be used as an objective to minimize in order to approximate $c^\dagger$. We use the Nelder-Mead simplex method to minimize $\Vert I_f-I_b\Vert$ at a chosen query value $I = I_\text {query} \in [0,I^*]$ (Figure 6). In contrast to the pulled front speed $c^*$, $c^\dagger$ depends on all parameters related to both direct and indirect transmission. This dependency is inherited from the potential function *V*(*I*), which in turn depends on *R*(*I*).

### Competition between pulled and pushed fronts

Pushed fronts may persist even as $I = 0$ loses its stability in the PDE (46), i.e. as *P* exceeds $(m+\delta )/\overline{\alpha }.$ During this transition, what was previously *the* stable eigendirection of $I = 0$ becomes the *most strongly attracting* direction. This direction can still connect the unstable direction of $I^*$, constituting a strongly heteroclinic connection (Powell 1997). The shooting method continues to estimate $c^\dagger$ in this case so long as $I_b$ is computed from the most strongly attracting direction.

When pulled fronts and (strongly heteroclinic) pushed fronts coexist, the pushed front will move faster and have a steeper profile, therefore setting the speed of invasion. To see why this is, let us solve the dispersion relation (50) for the shape parameter $\nu$:

56 $$\begin{aligned} \nu _{\pm } = \frac{1}{2\overline{\mu }}\left( -c \pm \sqrt{c^2 - 4\overline{\mu }R'(0)} \right) . \end{aligned}$$

The pulled front speed, $c^* = 2\sqrt{\overline{\mu }R'(0)}$, is the smallest positive *c* yielding real $\nu$ (i.e. non-oscillating fronts). This necessarily implies $c^\dagger \ge c^*$ since pushed fronts are assumed not to oscillate. As for steepness, note that $c^*$ maximizes $|\nu _+|$. Most fronts with speed $c > c^*$ will be shallower and less observable, since they connect along the weakly attracting direction with eigenvalue $\nu _+$. This is in accordance with the marginal stability conjecture (Avery and Scheel 2022). The single exception is the pushed front with speed $c^\dagger$, which connects along the strongly attracting direction and thus has steepness $|\nu _{-}(c^\dagger )|$. From (56) we see that $|\nu _-| > |\nu _+|$ for all *c*, so the pushed front is steeper. Whenever the strongly heteroclinic connection persists, pushed fronts will outcompete pulled fronts in this univariate version of our model.

## Results

Here we perform simulations to confirm theoretical results about invasion dynamics and expand our understanding of how landscape heterogeneity affects spread. First, we parameterize the model referring to data from Miller et al. (2006), adjusting some parameters to better represent WTD in WI. Then we use a gamma distribution of motilities to test plausible propagation behaviors of the homogenized model. Finally, we explore how CWD spread may be encouraged or inhibited using a two-habitat landscape model, and we use the homogenized equations to predict success or failure of potential interventions.

### Estimating Motility and Transmission Parameters

To determine the parameters related to disease dynamics – $\alpha , \beta , \gamma , \delta ,$ and *m* – a reasonable ballpark estimate can be achieved by referring to Miller et al. (2006), who recorded cumulate deaths of CWD-infected mule deer in confinement. These authors fit several models to the data and established the importance of indirect transmission, but did not include exposure thresholds. We introduce an ODE model modified from our infectious model with thresholds (1) to reflect the situation in the Miller study. In addition to the *S*, *I*, and *H* compartments, a cumulative CWD deaths component *C* is introduced. The deer in this study were kept in a pen (enclosure) much smaller than their native home ranges, so movement terms are neglected. Additionally, new susceptibles were added during the study, so an introduction term, *a*, is added. The ODE system modeling these factors is 57a57b57c57d Here *S*, *I*, *H* and *C* are functions of *T* (measured in years) only. The newly introduced parameter, *a*, measures the introduction rate for susceptible deer, with different values for the 1974-1984 and 1992-2001 experiments. Some parameters of this model may be fixed: $C(1974) = C(1992) = 0$ since no deer had died at the start of each experiment; $H(1974) = 0$ since the enclosure was not exposed to CWD prior to the study; $I(1992) = 0$ since the second cohort of deer were screened for CWD, specifically to demonstrate the infective capacity of environmental prion; and $m = 0.15$ from Miller et al. (2006). Futhermore, as explained in section 2, we assume $H_c = 1$ and other parameters are scaled accordingly.

With these assumptions, the model has a total of 7 model parameters and 4 initial conditions to fit. These values must be fitted on annual CWD cumulative death counts totalling only 21 data points, putting us at risk of overfitting. We remedy this somewhat by inferring more parameters from Miller et al. (2006). The authors do not provide initial conditions; however, they do disclose the structure and best-fit parameter values for several ODE models. Using this information, we can find the initial conditions *S*(1974), *I*(1974), and *S*(1992),  as well as the two values of *a*, that minimize the sum-squared error (SSE) of cumulative CWD deaths in the authors’ original models. We then fix these values in place when computing solutions to (57), which we use to fit $\alpha , \beta , \gamma , \delta , \tau ,$ and *H*(1992), again minimizing SSE. These minimizations are performed using the Nelder-Mead simplex method, available in matlab as fminsearch (Lagarias et al. 1998). Results can be found in Table 1.

These parameters were fit to data on mule deer kept in a small enclosure rather than free-ranging WTD in WI. We adjust some parameter values to translate between these scenarios. To begin with, contact rates were likely much higher in the enclosure than in the wild. Miller et al. (2006) suggest multiplying contact rates by 0.1 to reflect that individual mule deer ranges have 10 times less overlap in the wild than in the pen. The individual range for a mule deer is about $10\text {km}^2$ in Colorado (Miller et al. 2006); given WTD home ranges are roughly $2.5\text {km}^2$ in WI, we expect WTD contact rates to be roughly 4 times that of mule deer in the wild, and thus 0.4 times that of mule deer in the pen. Both contact rate estimates for $\alpha$ and $\beta$ are therefore multiplied by 0.4. Additionally, deer corpses were removed from the pen shortly after death. Tamgüney et al. (2009) estimate that the corpse of a CWD-infected individual deposits as much prion into the environment as the individual deposited over its lifespan. In the wild, where corpses remain, the total amount of prion deposited over a year should be approximately twice the amount deposited in the pen. Mathematically this is expressed $e^{\gamma \Delta t} = 2e^{{\widetilde{\gamma }}\Delta t}$, where ${\widetilde{\gamma }}$ is the excretion rate observed in the pen, $\gamma$ is the value in the wild, and $\Delta t$ is one year. Accordingly we let $\gamma = {\widetilde{\gamma }} + \ln 2$ be our estimate for excretion rates in the wild. The adjusted values of $\alpha ,$ $\beta ,$ and $\gamma$ are provided in Table 1.

**Table 1:** Parameter values for (1). The pen values are fitted directly to data from Miller et al. (2006), and adjusted values are explained in subsection 5.1. Parameters involving prion hazard are scaled using $H_c = 1$ infectious dose per square kilometer

Parameter	Definition	Units	Pen Value	Adjusted Value
$\alpha$	Direct transmissivity	km^2^/deer	0.0235	0.0094
$\beta$	Indirect transmissivity	km^2^/infectious dose	0.1141	0.0456
$\gamma$	Hazard excretion rate	infectious dose/deer	0.3306	1.0237
$\delta$	CWD mortality rate	1/year	0.4574	0.4574
*m*	Natural mortality rate	1/year	0.1500	0.1500
$\tau$	Hazard decay rate	1/year	2.0146	2.0146
$H_c$	Threshold density of hazard	infectious dose/km^2^	1	1

### Gamma-Distributed Motility

In this subsection, we assume the motility follows a gamma distribution (43). The homogenized parameters that result are $\overline{\mu } = 2/\lambda$, $\overline{\alpha }=2\alpha$, and *F*(*H*) as given in (44). We parameterize this motility by referring to Hefley et al. (2017), who fit a model of ecological diffusion by WTD in south-central WI. They found motility values ranging from 20 to $70 \text {km}^2/\text {yr}$ with a mean value around $30 \text {km}^2/\text {yr}$. To achieve this mean we set $\lambda = 0.1$. Note that $\overline{\mu }$ is not equal to the mean value of $\mu$ from the gamma distribution. Using $\overline{\mu },\overline{\alpha }, F(H)$, and other parameter values as in Table 1 gives a plausible description of CWD dynamics in WI.

Front speeds for the univariate PDE (46) are estimated using (53) for pulled fronts and the shooting method for pushed fronts. These estimates are compared to empirical measurements for simulated PDE solutions, which are computed using Strang splitting of diffusion and reaction operators, giving $2^\text {nd}$-order accuracy (MacNamara and Strang 2016). Diffusion is resolved using a Crank-Nicolson scheme, and the reaction problem is solved with the Runge Kutta method (LeVeque 2007). Simulations are initialized with an infectious population exceeding $I_\circ$, centered around the origin. The front location *f*(*T*) at a given time *T* is calculated as

58 $$\begin{aligned} f(T) = \min \{X\ :\ I(X,T) > 10^{-4}\}. \end{aligned}$$

**Fig. 7:**
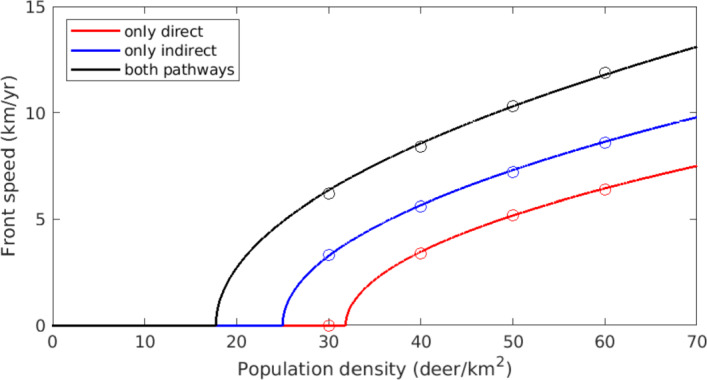
Speeds of advancing CWD fronts for a range of population densities. Parameter values are taken from Table 1, and $\mu$ follows a gamma distribution. Solid lines show front speed estimates, attained from (53) for pulled fronts and the shooting method described in subsection 4.3 for pushed fronts. The circles are empirically measured front speeds from numerical simulations of the homogenized univariate PDE (46). Red dots and lines correspond to the case where $\alpha > 0,\ \beta = 0$, so only pulled fronts can emerge. Blue elements correspond to $\alpha = 0,\ \beta > 0$, allowing only pushed fronts. Black elements have $\alpha , \beta > 0$, representing the most realistic estimate of CWD front speeds under our model. In this case fronts may be pushed or pulled. All front speed estimates in this final case were computed using the shooting method (Color figure online)

When fronts reach constant speed, their speed is measured by the slope of *f*. Our simulation measurements show excellent agreement with analytical predictions (Figure 7).

**Fig. 8:**
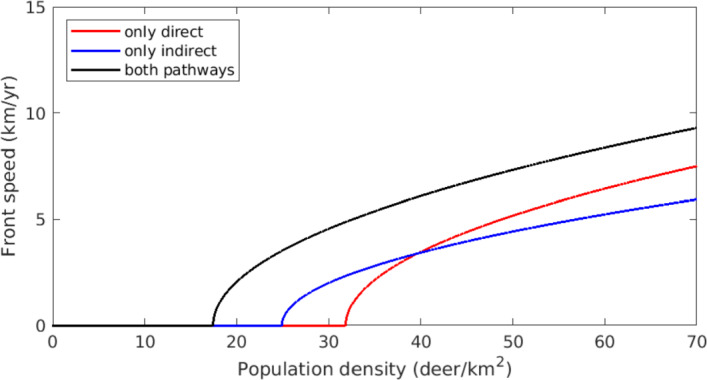
Speeds of advancing CWD fronts for solutions to (45), which does not assume a quasi-steady-state between infectives and prion. Compare with Figure 7. Since pushed front speed cannot be estimated without a univariate PDE, all speeds in this figure are measured from numerical simulation

To assess the relative impact of transmission pathways through pulled and pushed fronts, we consider three different cases: direct transmission only $(\alpha > 0, \beta = 0)$, so that only pulled fronts can develop; indirect transmission only $(\alpha = 0, \beta > 0)$, so that only pushed fronts can develop; and both pathways enabled $(\alpha , \beta > 0)$, which permits pulled and pushed fronts. In all three cases we treat the overall population density, *P*, as a bifurcation parameter. Notably, pushed fronts develop for lower population densities than pulled fronts in our model (7). As *P* increases past 32, pulled fronts grow in speed but always lag behind their pushed counterparts. This suggests the strongly heteroclinic connection persists even for large *P*. Conflicting behavior was found in McClure and Powell (2024), in which front speeds under the both-pathways scenario eventually merged with the pulled-front state. We suspect the quasi-steady-state (QSS) assumption prion hazard and infective population is responsible for this; if prion concentration lagged behind the arrival of infectives to an area, pushed front speeds are expected to drop. To test this, we numerically compute front speeds for the full system (45), which does not feature the QSS assumption. This system exhibits the same behavior seen in McClure and Powell (2024) (Figure 8), confirming the QSS is indeed responsible. Note the *P*-intercepts are the same for Figures 7 and 8, indicating that criteria for front existence are preserved when the QSS assumption is made.

### Alternating Habitat Scenarios

Homogenized model analysis can predict how disease will spread in various regions, potentially informing management decisions. To illustrate this, we consider a simplified landscape consisting of two alternating habitats. The first habitat has length $L_1$ and motility $\mu _1$. The second has length $L_2$ and motility $\mu _2 > \mu _1$, so it is less preferable to deer. These habitats alternate periodically, giving $\mu$ a period of $L_1 + L_2$. This allows us to compute homogenized quantities; for instance,

$$\begin{aligned} \overline{\mu }^{-1}&= \frac{1}{L_1+L_2}\int _{0}^{L_1+L_2} \frac{1}{\mu } \,\text {d}x \\&= \frac{1}{L_1+L_2}\left( \int _{0}^{L_1} \frac{1}{\mu _1}\,\text {d}x + \int _{L_1}^{L_2} \frac{1}{\mu _2}\,\text {d}x \right) = \frac{L_1\mu _2 + L_2\mu _1}{\mu _1\mu _2(L_1+L_2)}. \end{aligned}$$

The homogenized parameters $\overline{\mu }, \overline{\alpha }$ may be calculated using (10) and (28):

59 $$\begin{aligned} \overline{\mu } = \mu _1 \frac{M}{M-M\ell + \ell } \quad \text{ and }\quad \overline{\alpha } = \alpha \frac{M^2-M^2\ell + \ell }{(M-M\ell +\ell )^2}, \end{aligned}$$

where $M = \mu _2/\mu _1$, the ratio of motilities ($M > 1$), and $\ell = L_2/(L_1+L_2)$, the proportion of landscape consisting of undesirable habitat. The homogenized indirect transmission term, *F*(*H*), can be computed directly from (29) since there are only two values of $\mu$:

60 $$\begin{aligned} F(H) = p_1(H-H_1)^+ + p_2(H-H_2)^+, \end{aligned}$$

where

61 $$\begin{aligned} H_1&= H_c \frac{M-M\ell + \ell }{M}, \qquad H_2 &  = H_c \left( M -M\ell + \ell \right) ,\end{aligned}$$

62 $$\begin{aligned} p_1&= \frac{M^2-M^2\ell }{(M - M\ell + \ell )^2}, \qquad p_2 &  = \frac{\ell }{(M-M\ell + \ell )^2}. \end{aligned}$$

**Fig. 9:**
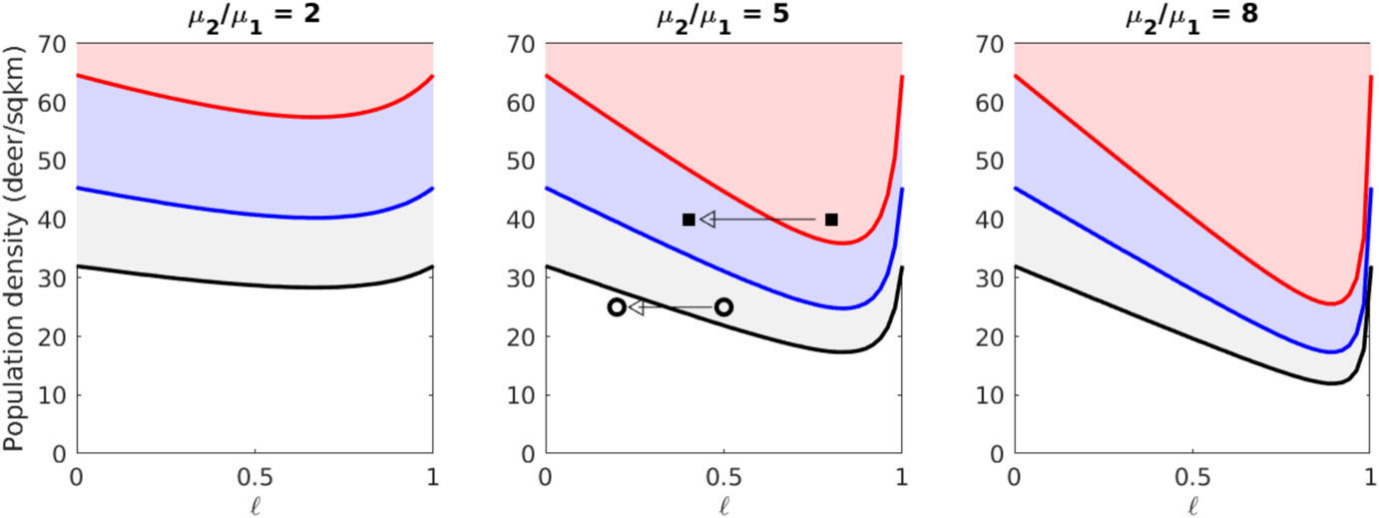
A map of possible fronts under various parameter combinations in a rapidly-periodic landscape of two habitats. Each plot considers the proportion of poorer habitat, $\ell$, and population density, *P*. Separate consideration is given to each combination of direct transmission, $\alpha$, and indirect transmission, $\beta$. Above the red curve, fronts can emerge when $\alpha > 0,\ \beta = 0$ (pulled). Above the blue curve, fronts emerge when $\alpha = 0,\ \beta > 0$ (pushed). Above the black curve, fronts emerge when $\alpha , \beta > 0$ (could be pushed or pulled). Different values of $M = \mu _2/\mu _1$ are used for each plot. In the middle plot, the circles represent the two landscapes simulated in Figure 10, illustrating a situation in which change of composition halts CWD spread. The squares represent the two landscapes simulated in Figure 11, in which one region supports pushed and pulled fronts but the other only pushed

Interestingly, $\overline{\alpha }$ and *F*(*H*) only depend on *M* and $\ell$. Thus whether a CWD front will propagate hinges on the relative quality and proportion of each habitat rather than on particular values of $\mu$. For a given *M* and $\ell$, we can calculate the minimum population density *P* that would allow front propagation in any of the cases considered in subsection 5.2 (Figure 9). Fronts fare better – they require smaller population densities and move faster for a given density – under a mixture of habitat types, regardless of ratio, than in a homogeneous environment. There is a mixture ratio that provides the most benefit to fronts; this ratio is always skewed toward a smaller amount of preferable habitat ($\ell > 0.5$) and increases with *M*.

Figure 9 can be used to predict conditions where CWD is expected to spread. For example, when $M = 5$ and $P = 25$, it appears that CWD fronts will progress when $\ell = 0.5$ but not when $\ell = 0.2$. To test this, we numerically simulate the non-homogenized system (1) subject to the constant population and QSS assumptions. The landscape in this simulation consists of two region with $\ell = 0.5$ and $\ell = 0.2$. CWD is able to spread through the first region but not the latter; thus, we have used homogenized parameters to predict the outcome in a non-homogenized simulation.

The transition from front-supporting to non-front-supporting regions is not the only one suggested by Figure 9. It also suggests transitions between the *types* of fronts carrying CWD. For example, consider the case where $M = 5$ and $P = 40.$ CWD is expected to spread when $\ell = 0.8$ or when $\ell = 0.4$; but in the former case the fronts could be pulled, and in the latter they must be pushed. This difference has tremendous impact when considering the implementation of a “firebreak” for CWD. Suppose that it is possible to haze or disturb deer in a region 20km wide, so that $\mu = 100\text {km}^2/\text {yr}$ in that region. Many individuals approaching the region would be reflected back the way they came, but some might make it to the other side. In a pulled front scenario, a few infectives crossing the break would be sufficient to restart the CWD front on the other side. By contrast, a pushed front requires a sufficient mass of infectives to cross, so a firebreak may be effective. To test this hypothesis, we numerically simulate the same PDE described above, this time with a landscape split into a pulled-front-supporting region and a pushed-front-supporting region. When a firebreak is implemented where pulled fronts exist, CWD makes its way across the break (11). When it is implemented in pushed front territory, infectives are effectively stopped at one side of the break.

**Fig. 10:**
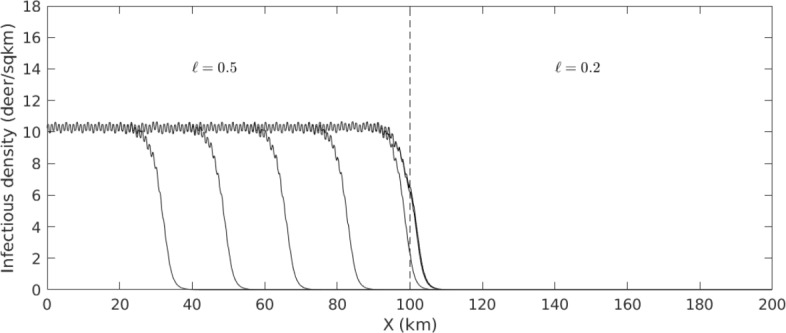
A CWD front failing to advance into territory with a lower proportion of unpreferred habitat. This experiment corresponds to the circles in Figure 9; accordingly, the population density is $P = 25$ and the domain is split into two regions with $\ell = 0.5$ and $\ell = 0.2$. Infectious individuals are initially centered around $x = 0$. The plots show infectious density *I*(*x*, *t*) for evenly-spaced values of *t*. The final three plots are all superimposed, indicating the front has come to a halt. Rapid variation in the front profiles arises from fine-scale alternation between two habitat types with different equilibrium densities (Color figure online)

**Fig. 11:**
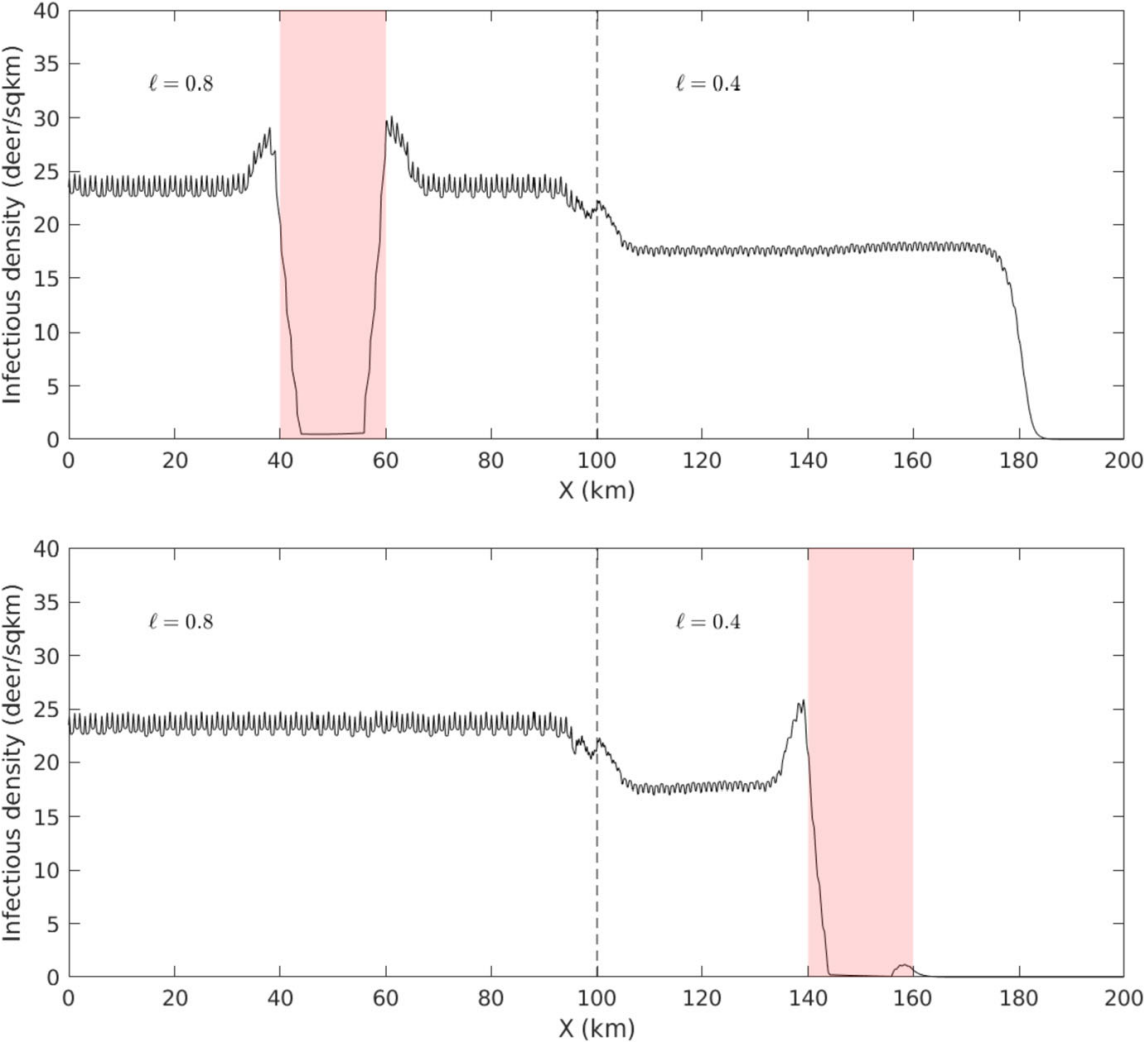
Two simulated implementations of a firebreak for CWD spread. These experiments correspond to the squares in Figure 9; thus the population density is $P = 40$ the transitions from a region with $\ell = 0.8$ to one with with $\ell = 0.4$. Infectives are initially concentrated around $x = 0$. Each figure shows $I(x,200\text {yrs}).$ The firebreak, indicated in red, is a 20km-wide region where $\mu = 100$. Top: the firebreak is placed in a region where pushed and pulled fronts propagate. Bottom: the firebreak is placed in a region supporting only pushed fronts. Rapid variation in the front profile arises from fine-scale alternation between two habitat types

## Discussion and Conclusion

In this paper, we constructed and homogenized a PDE model of CWD incorporating threshold-dependent environmental transmission and small scale landscape variation. Homogenization of this model resulted in a description of large-scale population densities modulated by local motility. Along the way, we developed a homogenized threshold transmission using an ergodic assumption on motility throughout the landscape. Calibrating this model using published data (Miller et al. 2006), it appears that pushed fronts spread CWD at lower population densities; pulled fronts require higher densities. Finally, we used a simple alternating landscape to illustrate that regions may differ in the types of fronts they support, which can be leveraged to inform allocation of resources to combat disease spread. In particular, we showed that homogenization accurately predicts locations where “firebreak" measures are suitable for halting pushed fronts.

The completely new expression for indirect transmission on large scales – represented by the function *F*(*H*) – is a major outcome of this paper. We find a closed form for *F* when motility is gamma-distributed, but the method we present can incorporate a wide variety of landscape complexities. As long as motility has a probability density $\rho$ with compact support and satisfying $\rho (0)=\rho '(0)=0,$ there will be some universal aspects shared with the graph of *F* shown in Figure 3. For *H* near 0, *F* has a tangent slope of zero, and in the large *H* limit *F* converges to a straight line. How the curve connects these two end conditions depends on the motility function. Thus landscape structure plays a critical role in indirect transmission for lower prion concentrations. This influence ultimately impacts the comparative success of pulled and pushed fronts across large regions.

The firebreak scenario considered in subsection 5.3 demonstrates the potential utility of homogenized modeling in designing disease mitigation strategies. In principle, one could analyze this scenario by first constructing a motility function with the break, then homogenizing the resultant PDE. This approach may yield an optimal spatial extent and degree of hazing for the break. Regardless, it is remarkable that an accurate prediction of mitigation success in the non-homogenized system was made just from analyzing the homogenized model sans-firebreak (Figure 9). The homogenized approach is also, of course, more computationally convenient. The alternating habitats are deliberately simple to prove the concept; a similar analysis could be performed on more complex landscapes.

For simplicity we have assumed that demographic transition parameters, such as transmissivity and excretion rates, are constant. In reality, habitat type influences more than just movement behavior. Deer engage in variably infective activities across habitats – for example, mineral licks are a known hotspot for environmental prion (Plummer et al. 2018). The homogenization technique as outlined in subsection 3.2 could accommodate spatial variability in several parameters to help investigate the impacts of variable behavior. For instance, suppose the direct transmissivity is given as a function $\alpha = \alpha ({{\textbf {x}}},{{\textbf {X}}})$ with the same quasi-periodic structure as $\mu$. Then the new homogenized transmissivity $\overline{\alpha }$ is still given by (28). If parameters related to *F*(*H*) are spatially varying, a closed form of *F* may still be found following the procedure in subsection 3.3. This would require a joint probability distribution for, e.g., $\mu$ and $\beta$. Additionally, our techniques would work on models with more detailed structure, such as the addition of age, sex or disease progress, which are known to influence CWD dynamics (Grear et al. 2010; Garlick et al. 2014; Tamgüney et al. 2009).

It remains a limitation of our model’s applicability that it was fit on data from mule deer in a pen. Additionally, it was found in McClure and Powell (2024) that long-distance dispersal of yearlings influences the spread of CWD, especially in the context of pushed fronts where critical densities of long-distance dispersers exacerbate spread. While including seasonal long-distance dispersal is beyond the scope of this paper, certainly it is possible. Now that we have an appropriate mathematical description for indirect transmission on large scales, we can use WI hunter surveillance data (Hefley et al. 2017) to determine transmission parameters directly. This knowledge could more definitely conclude whether pushed or pulled fronts drive the spread of CWD in WI.

We have focused on CWD in this paper, but there are several wildlife diseases in which indirect transmission plays a critical role. Varied examples include viral diseases like ebola and foot-and-mouth disease (Leroy et al. 2004; Bermejo et al. 2006; Mielke and Garabed 2020), fungal infectious like white nose syndrome (Cheng et al. 2021), and animal parasites with a latent larval stage (Bakke et al. 2002), to name a few. Where Allee or Allee-like effects are involved, we expect pushed front behavior. It has been shown that Allee-like dynamics can be induced through such varied mechanisms as differential vector preference (Hamelin et al. 2023) and sigmoidal infection rates (Regoes et al. 2002). These mechanisms may exhibit very different relationships between environment and transmission than the ones considered here. A similar homogenized-model analysis may be warranted in these cases. It may be the case that in another system, pulled and pushed fronts trade precedence depending on environmental parameters. Such an outcome could help researchers tailor specific mitigation strategies for different areas depending on their landscape composition.

More broadly, homogenized models of threshold exposure could find application in many contexts across ecology and biology. For example, ecotoxicology is a domain abundant with threshold exposures (Ritz 2010). A homogenized model of this type may be able to assess the large-scale effect of applying pesticides to the tops of crop plants across a whole farm, or the impact of unintentional toxin deposits on large landscapes where individuals have preferential habitats. In the realm of medicine, infections from surfaces are often thresholded (Joh et al. 2009), and the spread of disease from room to room may be likened to a homogenizable large-scale process on the scale of a hospital. Strong Allee effects have been analyzed heavily in ecology (e.g. Wang et al. 2011) and indeed homogenized in patchy landscape models (Maciel and Lutscher 2015); we hope that considering threshold exposure through this perspective may extend results to other domains that could benefit from it.

## Data Availability

All data is in the open literature and appropriately referenced.

## Appendix A Interval of integration for homogenized parameters

In practice, one cannot perform the homogenization procedure as outlined in subsection 3.2, since the quasi-period ${{\textbf {p}}} = (p_1,p_2)$ for the motility function $\mu$ (2) is unknown. This forbids us from averaging over the cell $\Omega _{{\textbf {X}}}$. Instead, we take averages over the set $[0,\varepsilon ^{-a}]^2$.

Suppose that *f* is quasi-periodic with quasi-period ${{\textbf {p}}}$. Adopting the notation $\left<f\right>_S = \frac{1}{|S|}\iint _S f \,\text {d}{{\textbf {x}}}$, our practical function averages are of the form

A1 $$\begin{aligned} \left<f \right>_{[0,\varepsilon ^{-a}]^2} = \frac{1}{\varepsilon ^{-2a}}\int _{0}^{\varepsilon ^{-a}}\int _{0}^{\varepsilon ^{-a}} f \,\text {d}x_1\text {d}x_2. \end{aligned}$$

The limits of this integral should tend to infinity as $\varepsilon \rightarrow 0$, ensuring that a sufficiently large region in ${{\textbf {x}}}$ is chosen to offset the increasing small-scale variation in *f*. Thus $a > 0$. On the other hand, performing the change of variables $X_1 = \varepsilon x_1$ and $X_2 = \varepsilon x_2$ yields

A2 $$\begin{aligned} \left<f \right>_{[0,\varepsilon ^{-a}]^2} = \frac{1}{\varepsilon ^{2-2a}}\int _{0}^{\varepsilon ^{1-a}}\int _{0}^{\varepsilon ^{1-a}}f \,\text {d}X_1\text {d}X_2. \end{aligned}$$

On the scale of ${{\textbf {X}}}$, the limits of this new integral should tend to zero as $\varepsilon \rightarrow 0$, so that averages are kept local with respect to the large scale. Thus $0< a < 1$ including the previous condition.

What error is incurred by performing the average defined in (A1) instead of the one from (26)? Following Garlick et al. (2011), let *M*, *N* be the largest positive integers such that $[0,Mp_1]\times [0,Np_2] \subset [0,\varepsilon ^{-a}]^2$, and furthermore let $R = [0,Mp_1]\times [0,Np_2].$ Denote by $R'$ the margins of $[0,\varepsilon ^{-p}]^2$ not covered by *R*, so that $R' = [0,\varepsilon ^{-p}]^2 \setminus R$. Then:

A3 $$\begin{aligned} \langle f \rangle _{[0,\varepsilon ^{-a}]^2} = \frac{1}{\varepsilon ^{-2a}}\left( \iint _R f\,\text {d}{{\textbf {x}}} + \iint _{R'}f\,\text {d}{{\textbf {x}}} \right) = \frac{|R|}{\varepsilon ^{-2a}}\left<f \right>_R + \frac{1}{\varepsilon ^{-2a}}\iint _{R'}f\,\text {d}{{\textbf {x}}}.\nonumber \\ \end{aligned}$$

Note that $\left<f\right>_R = \left<f\right>_{\Omega _{{\textbf {X}}}}$ due to the periodic structure of *f*. Additionally, the area of $R'$ is bounded above by $(M+1)p_1(N+1)p_2 - Mp_1Np_2 = (M+N+1)p_1p_2$, allowing for the inequality

A4 $$\begin{aligned} \left<f\right>_{[0,\varepsilon ^{-a}]^2}< \frac{|R|}{\varepsilon ^{-2a}}\left<f\right>_{\Omega _{{\textbf {X}}}} + \frac{(M+N+1)p_1p_2}{\varepsilon ^{-2a}}|f|_\text {max}. \end{aligned}$$

Suppose that $p_1, p_2,$ and $|f|_\text {max}$ are *O*(1). This implies that *M*, *N* are $O(\varepsilon ^{-a})$. The rightmost term in the previous equation would then be $O(\varepsilon ^{a})$. Finally, as $\varepsilon \rightarrow 0$, the ratio $|R| / \varepsilon ^{-2a}$ tends to 1, since the marginal region $R'$ shrinks to nothing. Putting it all together we have

A5 $$\begin{aligned} \left<f\right>_{[0,\varepsilon ^{-a}]^2} = \left<f \right>_{\Omega _{{\textbf {X}}}} + O(\varepsilon ^{a}). \end{aligned}$$

Of course, this analysis also applies to the inner product (20), which motivates the averages in the first place.

## Declarations

### Code Availability

Code is available upon reasonable request to the corresponding author.
